# The influence of stress factors on selected phenotypic and genotypic features of *Listeria monocytogenes* – a pilot study

**DOI:** 10.1186/s12866-023-03006-5

**Published:** 2023-09-16

**Authors:** Natalia Wiktorczyk-Kapischke, Ewa Wałecka-Zacharska, Jakub Korkus, Katarzyna Grudlewska-Buda, Anna Budzyńska, Kacper Wnuk, Eugenia Gospodarek-Komkowska, Krzysztof Skowron

**Affiliations:** 1 Department of Microbiology, Ludwik Rydygier Collegium Medicum in Bydgoszcz, Nicolaus Copernicus University, Toruń, Poland; 2 Department of Food Hygiene and Consumer Health, Wrocław University of Environmental and Life Sciences, Wrocław, Poland; 3 Department of Theoretical Foundations of Biomedical Sciences and Medical Computer Science, Ludwik Rydygier Collegium Medium in Bydgoszcz, Nicolaus Copernicus University, Toruń, Poland

**Keywords:** *Listeria monocytogenes*, Gene expression, Stress, environmental stress, Antibiotics, Biofilm, Motility, qPCR, Growth rate

## Abstract

**Background:**

*Listeria monocytogenes* are Gram-positive rods, widespread in the environment due to their wide tolerance to changing conditions. The apilot study aimed to assess the impact of six various stresses (heat, cold, osmotic, acid, alkali, frozen) on phenotypic features: MIC of antibiotics (penicillin, ampicillin, meropenem, erythromycin, co-trimoxazole; gradient stripes), motility, ability to form a biofilm (crystal violet method) and growth rate (OD and quantitative method), expression level of *sigB* (stress induced regulator of genes), *agrA, agrB* (associated with biofilm formation) and *lmo2230, lmo0596* (acid and alkali stress) (qPCR) for three strains of *L. monocytogenes*.

**Results:**

Applied stress conditions contributed to changes in phenotypic features and expression levels of *sigB*, *agrA*, *agrB*, *lmo2230* and *lmo0596*. Stress exposure increased MIC value for penicillin (ATCC 19111 - alkaline stress), ampicillin (472CC - osmotic, acid, alkaline stress), meropenem (strains: 55 C - acid, alkaline, o smotic, frozen stress; 472CC - acid, alkaline stress), erythromycin (strains: 55 C - acid stress; 472CC - acid, alkaline, osmotic stress; ATCC 19111 - osmotic, acid, alkaline, frozen stress), co-trimoxazole (strains: 55 C - acid stress; ATCC 19111 - osmotic, acid, alkaline stress). These changes, however, did not affect antibiotic susceptibility. The strain 472CC (a moderate biofilm former) increased biofilm production after exposure to all stress factors except heat and acid. The ATCC 19111 (a weak producer) formed moderate biofilm under all studied conditions except cold and frozen stress, respectively. The strain 55 C became a strong biofilm producer after exposure to cold and produced a weak biofilm in response to frozen stress. Three tested strains had lower growth rate (compared to the no stress variant) after exposure to heat stress. It has been found that the *sigB* transcript level increased under alkaline (472CC) stress and the *agrB* expression increased under cold, osmotic (55 C, 472CC), alkali and frozen (472CC) stress. In contrast, *sigB* transcript level decreased in response to acid and frozen stress (55 C), *lmo2230* transcript level after exposure to acid and alkali stress (ATCC 19111), and *lmo0596* transcript level after exposure to acid stress (ATCC 19111).

**Conclusions:**

Environmental stress changes the ability to form a biofilm and the MIC values of antibiotics and affect the level of expression of selected genes, which may increase the survival and virulence of *L. monocytogenes*. Further research on a large *L. monocytogenes* population is needed to assess the molecular mechanism responsible for the correlation of antibiotic resistance, biofilm formation and resistance to stress factors.

## Introduction

*Listeria monocytogenes* are Gram-positive, facultative anaerobic, non-spore-forming bacteria widespread in the environment (soil, water, sewage, animal feed, rotting vegetation, food) [1, 2]. These bacteria are the etiological agent of listeriosis. Pregnant women, the elderly and people with immunosuppression are particularly at risk of infection. The mortality rate of listeriosis patients is around 30% [3]. Food is the main source of rods for humans, e.g., meat, fish, unpasteurized milk products, raw fruit and vegetables and ready-to-eat (RTE) products [4]. According to the European Food Safety Authority (EFSA) report, in 2021, 2,183 cases of listeriosis were confirmed in the European Union [5]. The monitoring of *L. monocytogenes* within environment and food processing facilities is an important aspect of public health protection.

A characteristic feature of *L. monocytogenes* is the ability to survive and adapt to unfavorable environmental conditions (including low and high temperatures, a wide range of pH and salinity, low water activity) [6, 7]. Its adaptability makes this pathogen difficult to eradicate within food processing facilities, which can lead to food cross-contamination [8]. In addition, *L. monocytogenes* can form a biofilm on various surfaces (biotic and abiotic), e.g., stainless steel, polypropylene, rubber (surfaces often found in the food industry) [9, 10]. According to Di Cicio et al. [11], biofilm enables the survival of bacteria in the food industry. The biofilm structure protects *L. monocytogenes* from extreme environmental conditions, antimicrobials and disinfectants [12–14].

Another critical and global public health problem are antibiotic-resistant bacteria, including *L. monocytogenes* [15, 16]. Primary therapy for patients with listeriosis includes penicillin, ampicillin with gentamicin or vancomycin, co-trimoxazole and erythromycin for patients intolerant to β-lactam antibiotics [16, 17]. Threlfall et al. [18] have demonstrated high antibiotic resistance among *L. monocytogenes* strains. Environmental stress may also contribute to the development of resistance to a range of antibiotics [19]. Al-Nabulsi et al. [20] have noted that exposure to selected stress conditions commonly present in food processing increased minimum inhibitory concentration (MIC) of selected antibiotics. Antibiotic resistance acquisition under stress conditions, especially in food isolates, can lead to failure of the therapeutic treatment of listeriosis.

*L. monocytogenes* triggers several mechanisms to survive in the harsh environment. One is associated with the alternative sigma B factor (σ^B^) (general stress response). σ^B^ has been shown to contribute to *L. monocytogenes* survival under different stresses, e.g., cold, acid, osmotic, heat and oxidative. In addition, this alternative sigma factor regulates or putatively regulates expression of genes encoding putative efflux pumps, penicillin binding proteins, autolysins, and cell wall-related proteins [21]. σ^B^ also affects stress-induced activation of virulence genes [22] and biofilm formation ability [23]. Lee and Wang [24] have shown that genes related to the synthesis of extracellular polymeric substances (EPS) (considered as the elemental component determining the physicochemical properties of biofilm) are regulated by the Agr quorum sensing (QS) system.

Based on current knowledge, each strain of *L. monocytogenes* can be considered potentially pathogenic to humans. The virulence among *L. monocytogenes* population is heterogenic and strain-dependent [25–27]. To elucidate the virulent potential of *L. monocytogenes*, it is essential to simultaneously assess changes in phenotypic traits and gene expression levels [28].

This pilot study aimed to assess the impact of selected stress conditions (heat (20 min, 55 °C, cold (7 d, 4 °C), osmotic (4.5% NaCl, 3 h, 37 °C), acid (pH 5, 80 min, 37 °C), alkali (pH 8, 80 min, 37 °C) and frozen (24 h, -20 °C)) on selected phenotypic features (MIC of selected antibiotics, motility, ability to form a biofilm) and growth rates (in a no stress and stress-exposed bacteria) of three *L. monocytogenes* strains. In addition, the expression level of selected genes associated with general stress response (*sigB*), biofilm formation ability (*agrA, agrB*) and acid and alkali stress (*lmo2230*, *lmo0596*) was determined.

## Materials and methods

### Material

The investigated bacterial isolates are *L. monocytogenes* from the collection of the Department of Microbiology of Ludwik Rydygier Collegium Medicum in Bydgoszcz, Nicolaus Copernicus University in Toruń. The examined strains included: the strain isolated from clinical material (55 C), the strain isolated from cold cuts (472CC) and the reference strain *L. monocytogenes* ATCC 19111. Table 1 presents characteristics of the examined strains. The isolates were stored in brain-heart infusion broth (BHI, Merck) with 15.0% glycerol (Avantor) at -80 °C until the beginning of the research.

**Table 1:** Initial characteristics of the examined *L. monocytogenes* strains

Strain number	Isolation source	Serogroup	Antibiotic resistance profile*	Presence of virulence genes
55 C	Clinical material (cerebro-spinal fluid)	1/2a-3a	R: - S: P, AMP, MEM, E, STX	LIPI-1, *inlA, inlB*, *fbpA, iap*
472CC	Cold cuts	1/2a-3a	R: - S: P, AMP, MEM, E, STX	LIPI-1, *inlA, inlB*, *fbpA, iap*
ATCC 19111 **	Reference strain (isolated from poultry)	Serotype 1/2a	R: - S: P, AMP, MEM, E, STX	LIPI-1, *inlA*, *inlB*, *fbpA*, *iap*

C – clinical; CC – cold cuts; ATCC - American Type Culture Collection; * - on the basis of the disc-diffusion method; ** - data from sheet information; R – resistance; S – sensitive; P – penicillin; AMP – ampicillin; MEM – meropenem; E – erythromycin; STX – co-trimoxazole (sulfamethoxazole + trimethoprim); LIPI – 1 – *Listeria* Pathogenicity Island 1 (containing genes: *prfA*, *plcA*, *hlyA*, *mpl*, *actA*, *plcB*)

### Preparation of ***Listeria monocytogenes*** strains for research

The examined strains (from freezing) were plated on Columbia agar with 5.0% sheep blood (CAB, Graso) (24 h, 37 °C) using streak plate method. Then, a single colony (cluster of bacterial cells derived from the same mother cell) of each strain (individually) was inoculated on CAB (24 h, 37˚C) using streak plate method. After the incubation period, 3 single colonies of each strain (individually) were seeded into 10 ml of Tryptic Soy Broth (TSB, Graso) (20 h, 37˚C).

### Impact of selected stress factors on ***Listeria monocytogenes***

#### Post-stress procedure

After contact with a stress factor, bacteria were centrifuged (5 min, 12,000 x g), and the supernatant was removed. The resulting pellet was washed with TE (Eur_X_) buffer, centrifuged again (5 min, 12,000 x g). The supernatant was removed, and the remaining pellet was used for further studies. At the same time, a suspension not subjected to stress factors was prepared.

#### Heat stress

The bacterial suspension was placed in a thermoblock (Eppendorf) and exposed to high temperature (55˚C, time: 20 min), followed by the “post-stress procedure”.

#### Cold stress

The bacterial culture was placed at 4˚C for 7 d, followed by the “post-stress procedure”.

#### Osmotic stress

Bacteria were exposed to 4.5% NaCl. Briefly, bacterial suspension was mixed with an equal volume of TSB (Graso) containing 9.0% NaCl (Avantor), and then incubated for 3 h at 37˚C (time based on: [29]), followed by the “post-stress procedure”.

#### Acid and alkaline stress

The bacterial suspension was combined with an equal volume of TSB (Graso) with the appropriate pH concentration (5 (acid) and 8 (alkali)), and then incubated for 80 min at 37˚C (time based on: [30]), followed by the “post-stress procedure”.

#### Frozen stress

The bacterial suspension was placed at -20˚C for 24 h. After this time, the culture was removed from freezer and left for 3 h at room temperature (23 °C) until completely thawed, followed by the “post-stress procedure”.

### Evaluation of the impact of stress factors on selected phenotypic features of ***Listeria monocytogenes***

#### Evaluation of the ability to form a biofilm

The biofilm determination was carried out in accordance with the methodology proposed by Kwiecińska-Piróg et al. [31]. The resulting pellet was dissolved in Mueller Hinton Broth (MHB, Becton Dickinson) to give optical density of 0.5 McFarland. Then, 20 μl of each suspension were placed in wells of 96-well plates (Profilab) (in triplicate), and 180 μl of MHB medium (10-fold dilution of the suspension) was added. For the negative control 200 μl of sterile MHB medium was used. The plates were incubated in a humid chamber (24 h, 37 °C). Next, the suspensions were removed by pipetting and the wells were washed three times with sterile distilled water. The plates were air-dried at 37 °C for 20 min. Then, 200 μl of methanol (POCH) was added and the plates were shaken (400 RPM) at room temperature for 20 min. Next, ethanol was removed, 200 μl of 0.1% crystal violet (POCH) solution was added and the plates were shaken (400 RPM) at room temperature for 20 min. Then, crystal violet was removed and wells were washed with water until colorless washings. The plates were allowed to evaporate and then 200 μl of methanol (POCH) was added. After 5-minute shaking (400 RPM, at room temperature) absorbance at 570 nm (Abs_570_) was read in a Synergy™HT multidetection reader (BIO-TEK). The average Abs_570_ value, obtained from triplicates for each strain, was determined in the Gen5 3.11 program. Comparing the absorbance of the strains (A) with the absorbance of the negative control (K-), it was possible to classify the strains as strongly, moderately and weakly biofilm-forming, according to the method described by Stepanović et al. [32]. Cut-off values were established: K- < A ≤ 2 × K- - weak biofilm producer; 2 × K- < A ≤ 4 × K- - moderate biofilm producer, 4 × K-< A - strong biofilm producer.

#### Evaluation of the minimum inhibitory concentration (MIC) of selected antibiotics

The pellet was dissolved in sterile physiological saline (Polpharma) to obtain a suspension with an optical density of 0.5 McFarland. The prepared suspension was plated on Mueller Hinton Agar with 5.0% horse blood and β-NAD (MHF, Graso) and then gradient strips with antibiotics, i.e., penicillin (0.016-256 μg/ml) (Liofilchem), ampicillin (0.016-256 μg/ml) (Liofilchem), meropenem (0.002-32 μg/ml) (Liofilchem), erythromycin (0.016-256 μg/ml) (Liofilchem) and trimethoprim*/sulfamethoxazole (1/19) (co-trimoxazole) (0.002-32* μg/ml) (Liofilchem) were applied. After 20-h incubation at 35 °C MICs (based on the eclipse-shaped inhibition zone) were determined. The results were interpreted in accordance with EUCAST v. 13.0 recommendations [33].

#### Motility assessment

To assess the motility, 0.4% agar (BTL) was stabbed with a needle-type loop (approximately 1 cm) with the bacterial suspension of 0.5 McFarland (prepared like above) and incubated at 22 °C for 48 h. In the case of motility about 0.5 cm below the surface of the agar an umbrella-like growth was observed after 24 and 48 h (based on: [34]).

#### Growth rates

Growth rates were evaluated for the no stress and stress-exposed variants.

After “post-stress procedure”, bacterial pellets were dissolved in TSB to 0.5 McF. Then, the suspensions were diluted 100-fold in TSB, 100 μl of each diluted suspension were placed in 96-well plates (Profilab) (in triplicate), and 100 μl of TSB medium was added. For the negative control 100 μl of sterile TSB medium and 100 μl of sterile Phosphate-buffered saline (PBS, BTL) was used. The plates were incubated in a humid chamber (24 h, 37 °C). Absorbance at 600 nm (Abs_600_) was read in a Synergy™HT multidetection reader (BIO-TEK) at the respective time points: 0, 2, 4, 6, 8, 10 and 24 h. The average Abs_600_ value, obtained from triplicates for each strain, was determined in the Gen5 3.11 program.

To assess the number of bacteria, the diluted suspension was mixed with an equal volume of sterile TSB (final volume: 10 ml). Bacteria were incubated for 24 h at 37 °C. At the designated time points (0, 2, 4, 6, 8, 10, and 24 h), 0.5 ml of the suspension was collected, followed by a serial 10-fold dilutions in PBS. Two selected dilutions were plated (100 μl) on trypticase soy agar (TSA, Graso) (in duplicate) and after 24 h (37˚C), grown colonies were counted and presented as log CFU (colony forming unit)/ml.

### Evaluation of expression of selected genes after exposure to stress factors

#### RNA isolation

The stressed cells were resuspended in 0.1 M Tris-HCl of pH 7.4 (Sigma-Aldrich) + lysozyme (10 mg/ml, Eur_X_) + proteinase K (~ 20 mg/ml, Thermo Fisher Scientific) + 10% SDS (Sigma-Aldrich) and incubated at 37 °C, for 30 min. Then the suspension was placed at 80 °C for 5 min (thermoblock). Next, 1 ml TRI reagent (Sigma-Aldrich) was added, and the suspension was placed at -80 °C for 20 min. Then, 200 μl of PURE chloroform (POCH) was added, and the samples were left for 5 min at room temperature (23 °C) for phase separation. After centrifugation (15 min, 12,000 x g, 4 °C) the upper phase (500 μl) was transferred to a new eppendorf tube, and an equal volume of isopropyl alcohol (POCH) was added. After 20-min incubation at -20 °C, samples were centrifuged (10 min, 12,000 x g, 4 °C), the supernatant was removed, and 1 ml of 70% ethanol (POCH) was added. After 20-min incubation at -20 °C, samples were centrifuged (5 min, 7,600 x g, 4 °C), and the precipitate was allowed to dry at room temperature (23 °C) for 7 min and then dissolved in 20 μl of RNase-free (Eur_X_) water. RNA was digested with DNase (2U; A&A Biotechnology) (37˚C, 90 min). RNA was stored at -80 °C until qPCR was performed.

#### Reverse transcription and real-time PCR

cDNA synthesis was performed on 1 μg of RNA using the iScript™ cDNA Synthesis Kit (Bio-Rad) following the manufacturer’s instructions. cDNA was stored at -20 °C. The relative amounts of *sigB*, *agrA*, *agrB*, as well as *lmo2230* and *lmo0596* (for acid and alkali stress) transcripts were determined using the CFX96 Optical System (BioRad, Warsaw, Poland). Table 2 presents primer sequences (based on: [35–38]). For normalization of cDNA amount, the housekeeping gene *gap* was used [35]. Each PCR was performed in duplicate from two independent RNA preparations. PCR was performed in a mixture containing: 1 μl cDNA, 500 nM *gap*, *sigB*, *agrA*, *agrB*, *lmo2230* and *lmo0596* primers, iTaq Universal SYBR Green Supermix (Bio-Rad) and water (Eur_X_). The amplification consisted of 40 cycles of: 30 s denaturation at 95˚C, annealing for 30 s at 60˚C, and an elongation for 45 s at 72˚C, preceded by an initial denaturation at 95˚C for 3 min. To determine the degree of RNA contamination with genomic DNA for each sample No-RT (no reverse transcription) controls were included. For each primer PCR efficiency was determined using a serial 10-fold dilutions of the template (genomic DNA). Determined efficiencies were included when calculating relative transcript levels according to Pfaffl [39].

**Table 2:** Primer sequences used in study

Gene	Primer (reverse)	Primer (forward)	References
*gap*	TGGTGTTGTTGAAGGTCTAATG	GCAGCTCCGTCTAATTTACC	[35]
*sigB*	TGGTGTCACGGAAGAAGAAG	TCCGTACCACCAACAACATC	[36]
*agrA*	CGGGTACTTGCCTGTATGAA	TGAATAGTTGGCGCTGTCTC	
*agrB*	CGGCAGACACAGAAAGTTTG	TGCGAATGGTATTAGCAACG	[37]
lmo2230*	CTGAACTAGGTGAATAAGACAAAC	CATATTCGAAGTGCCATTGC	[38]
lmo0596*	CCCACATACCGAAAAGTAATACGAG	GGGTACTAGCTGACGGAATTTTATC	

* - for acid and alkali stress

### Statistical analysis

The statistical analysis was performed in Excel (Microsoft). A Welch’s t-test with Bonferroni correction was used to determine statistical differences between experimental groups and “no stress” (reference) group with significance level set at alpha = 0.05.

## Results

### Biofilm formation ability after exposure to stress factors

All examined strains were able to form biofilm, both before and after exposure to stress factors (Tables 3, 4 and 5). Strain 55 C, a moderate biofilm producer, became strong and weak biofilm-former after exposure to cold and frozen stress, respectively (Table 3). Another strain 472CC significantly increased biofilm production (from moderate to strong) after exposure to all stress factors except heat and acid (Table 4). The last strain ATCC 19111, classified as a weak biofilm-former, produced moderate biofilm in response to all stresses, excluding frozen and cold shock (Table 5).

**Table 3:** The ability to form a biofilm by the strain 55 C in different stress variants

Experiment variant	Average absorbance value (minus blank)	Standard deviation	Coefficient of variation [%]	Strain classification as a producer of biofilm
basic variant (no stress)	0.213	0.009	4.44	moderate
heat stress	0.163	0.020	12.42	moderate
cold stress	0.311	0.015	4.92	strong
osmotic stress	0.162	0.002	1.52	moderate
acid stress	0.137	0.001	0.40	moderate
alkali stress	0.128	0.008	6.10	moderate
frozen stress	0.106	0.003	2.87	weak

**Table 4:** The ability to form a biofilm by the strain 472CC in different stress variants

Experiment variant	Average absorbance value (minus blank)	Standard deviation	Coefficient of variation [%]	Strain classification as a producer of biofilm
basic variant (no stress)	0.183	0.014	7.88	moderate
heat stress	0.280	0.008	2.78	moderate
cold stress	0.342	0.014	4.02	strong
osmotic stress	0.368	0.026	6.98	strong
acid stress	0.240	0.047	19.70	moderate
alkali stress	0.336	0.004	1.13	strong
frozen stress	0.324	0.011	3.53	strong

**Table 5:** The ability to form a biofilm by the strain ATCC 19111 in different stress variants

Experiment variant	Average absorbance value (minus blank)	Standard deviation	Coefficient of variation [%]	Strain classification as a producer of biofilm
basic variant (no stress)	0.067	0.003	4.82	weak
heat stress	0.180	0.014	7.93	moderate
cold stress	0.075	0.003	3.37	weak
osmotic stress	0.164	0.010	5.88	moderate
acid stress	0.138	0.001	0.42	moderate
alkali stress	0.121	0.008	6.45	moderate
frozen stress	0.110	0.000	0.00	weak

### Evaluation of the minimum inhibitory concentration (MIC) of selected antibiotics

The results of the MIC value assessment showed that all examined strains were sensitive to antibiotics used, both in the basic variant and after exposure to stress factors (Tables 6, 7 and 8). Stress factors, however, changed MIC values of selected antibiotics. In the case of strain No. 55 C, heat, acid, and frozen stress decreased the MIC of penicillin (from 0.19 to 0.125 μg/ml). Heat, osmotic and frozen stress reduced the MIC of ampicillin (from 0.19 to 0.125 μg/ml). In turn, acid, alkaline, osmotic and frozen stress increased the MIC of meropenem (from 0.064 to 0.094 μg/ml), and heat stress decreased this value (to 0.047 μg/ml). A decrease of erythromycin MIC (from 0.25 to 0.125 μg/ml) and co-trimoxazole MIC (from 0.32 to 0.23 μg/ml) was observed in response to heat and heat and osmotic stress, respectively. In turn, alkaline and acid stress elevated the MIC values of erythromycin (0.5 μg/ml) and co-trimoxazole (0.047 μg/ml), respectively (Table 6).

**Table 6:** Results of the MIC value assessment of selected antibiotics for strain 55 C

Experiment variant	Penicillin [μg/ml]	Ampicillin [μg/ml]	Meropenem [μg/ml]	Erythromycin [μg/ml]	Co-trimoxazole [μg/ml]
basic variant (no stress)	0.19	0.19	0.064	0.25	0.032
heat stress	0.125	0.125	0.047	0.125	0.023
cold stress	not marked				
osmotic stress	0.19	0.125	0.094	0.25	0.023
acid stress	0.19	0.19	0.094	0.25	0.047
alkali stress	0.125	0.19	0.094	0.50	0.032
frozen stress	0.125	0.125	0.094	0.25	0.032

marked green - a decrease in relation to the basic variant; marked red - increase in relation to the basic variant; marked white - no changes

**Table 7:** Results of the MIC value assessment of selected antibiotics for strain 472CC

Experiment variant	Penicillin [μg/ml]	Ampicillin [μg/ml]	Meropenem [μg/ml]	Erythromycin [μg/ml]	Co-trimoxazole [μg/ml]
basic variant (no stress)	0.19	0.125	0.064	0.38	0.023
heat stress	0.125	0.094	0.047	0.19	0.016
cold stress	not marked				
osmotic stress	0.19	0.19	0.064	0.50	0.012
acid stress	0.19	0.19	0.094	0.50	0.012
alkali stress	0.19	0.19	0.094	0.50	0.012
frozen stress	0.19	0.094	0.064	0.38	0.012

marked green - a decrease in relation to the basic variant; marked red - increase in relation to the basic variant; marked white - no changes

**Table 8:** Results of the MIC value assessment of selected antibiotics for strain ATCC 19111

Experiment variant	Penicillin [μg/ml]	Ampicillin [μg/ml]	Meropenem [μg/ml]	Erythromycin [μg/ml]	Co-trimoxazole [μg/ml]
basic variant (no stress)	0.047	0.047	0.032	0.125	0.023
heat stress	0.047	0.032	0.032	0.125	0.016
cold stress	not marked				
osmotic stress	0.047	0.032	0.032	0.19	0.032
acid stress	0.047	0.047	0.023	0.25	0.032
alkali stress	0.064	0.047	0.032	0.25	0.032
frozen stress	0.047	0.047	0.032	0.25	0.023

marked green - a decrease in relation to the basic variant; marked red - increase in relation to the basic variant; marked white - no changes

In the case of strain No. 472CC, heat stress reduced the MIC of penicillin (from 0.19 to 0.125 μg/ml). Heat and frozen stress decreased the MIC of ampicillin (from 0.125 to 0.094 μg/ml), while acid, alkaline and osmotic stress increased this value (to 0.19 μg/ml). Acid and alkaline stress elevated MIC values (from 0.64 to 0.94 μg/ml) of erythromycin (from 0.38 to 0.50 μg/ml), and meropenem (from 0.64 to 0.94 μg/ml), while heat exposure reduced these values (to 0.047 μg/ml for meropenem and to 0.19 μg/ml for erythromycin). Also, osmotic stress contributed to increased MIC of erythromycin. On the other hand, co-trimoxazole MIC values decreased in response to all tested stress variants (Table 7). Heat stress decreased MIC values of all tested antibiotics in both the clinical strain and the strain isolated from cold cuts (Tables 6 and 7).

In the case of strain ATCC 19111, an increase in the MIC of penicillin (from 0.47 to 0.64 μg/ml) was observed after exposure to alkaline stress. Heat and osmotic shock reduced ampicillin MIC (from 0.47 to 0.32 μg/ml). Low pH decreased MIC of meropenem (from 0.32 to 0.23 μg/ml), whereas acid, alkaline, and frozen stress exposure elevated erythromycin MIC (from 0.125 to 0.25 μg/ml). In the case of co-trimoxazole, osmotic, acid, and alkaline stress increased MIC values (from 0.23 to 0.32 μg/ml), while heat stress caused a decrease (to 0.16 μg/ml) (Table 8).

### Motility after exposure to stress factors

All examined *L. monocytogenes* strains, both subjected and not subjected to all stress factors, were motile after 48 h incubation. Together with incubation time, (in mm) the characteristic “umbrella” elongated (Table 9). The ATCC 19111 strain, after exposure to heat stress, did not show motility after 24 h (0 mm). However, after 48 h, this strain was motile (Table 9).

**Table 9:** The size of the movement zone [mm] in the form of a characteristic “umbrella” among the tested strains

Strain number	Time point [h]	Size of motility zone [mm]						
		No stress	After acid stress	After alkali stress	After osmotic stress	After heat stress	After cold stress	After frozen stress
55 C	24	19	13	11	14	9	15	11
	48	24	22	20	25	23	21	25
472CC	24	22	13	18	13	11	15	13
	48	25	23	30	24	25	22	25
ATCC 19111	24	20	18	16	10	0	8	5
	48	28	26	23	19	27	18	18

C – clinical; CC – cold cuts; ATCC - American Type Culture Collection

### Growth rates

**Fig. 1:**
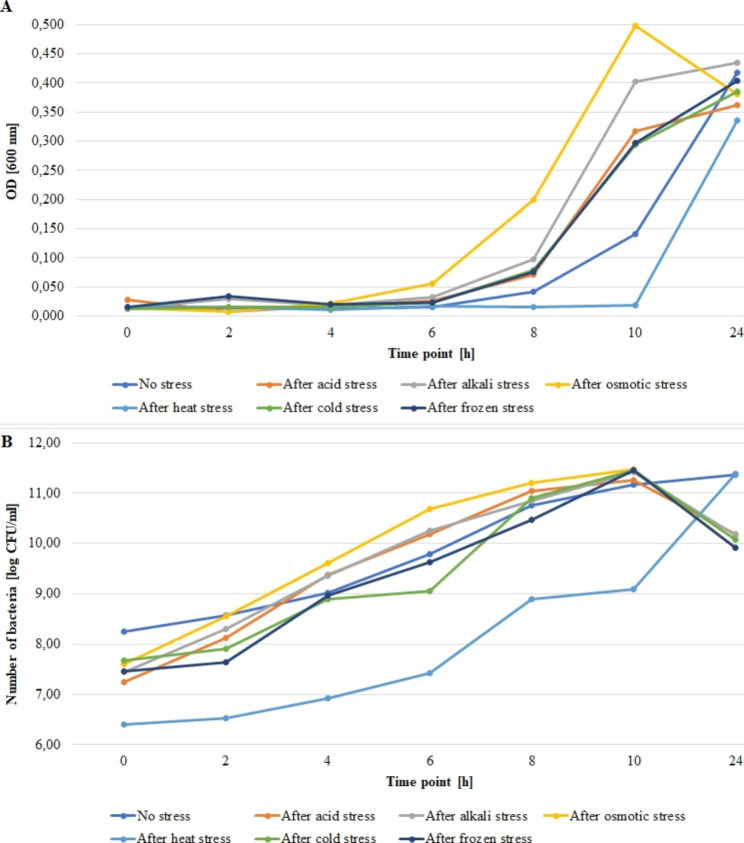
Growth rates for the no-stress and post-stress variants for strain 55 C. (**A**) OD value, (**B**) number of bacteria

**Fig. 2:**
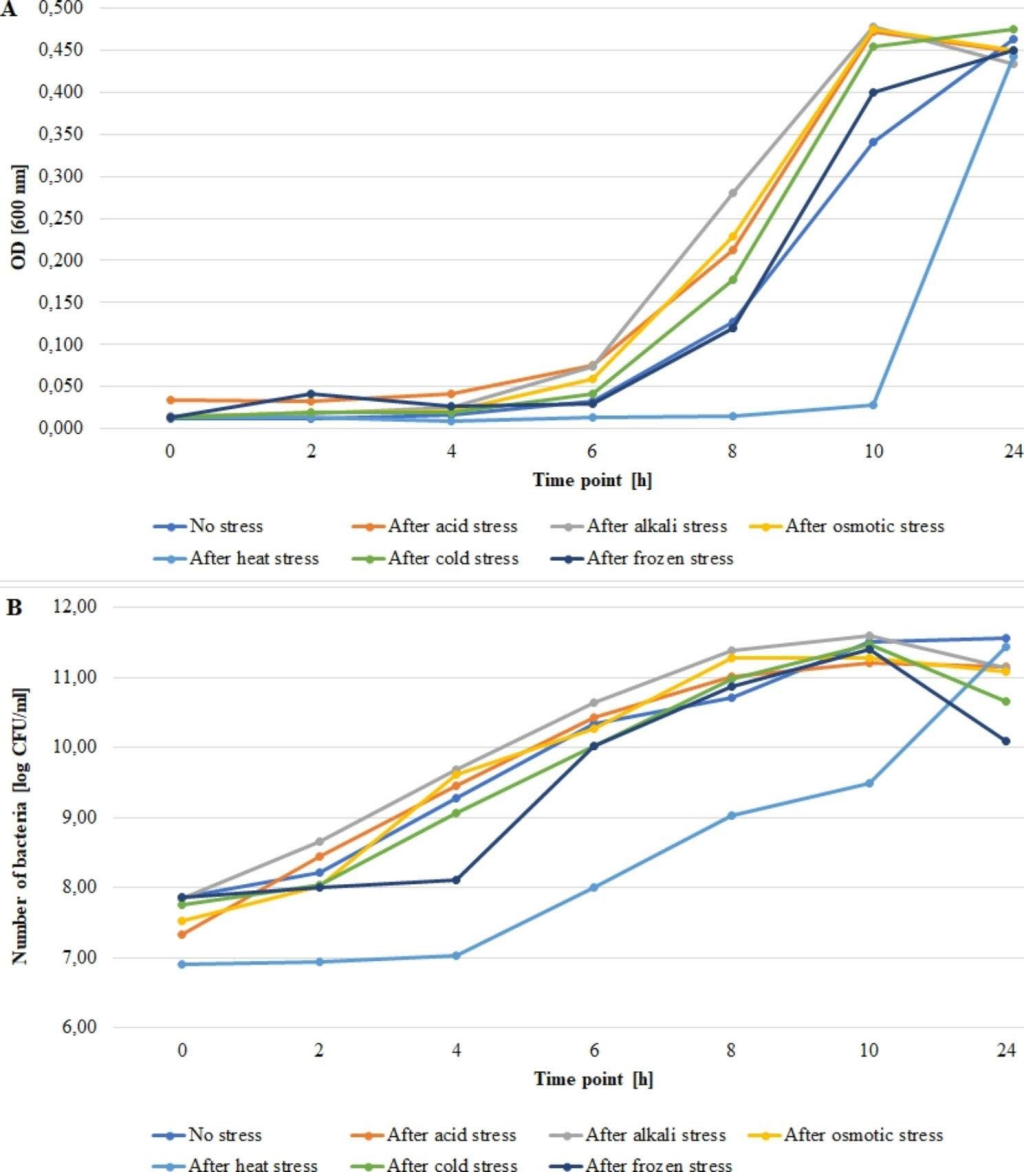
Growth rates for the no-stress and post-stress variants for strain 472CC. (**A**) OD value, (**B**) number of bacteria

**Fig. 3:**
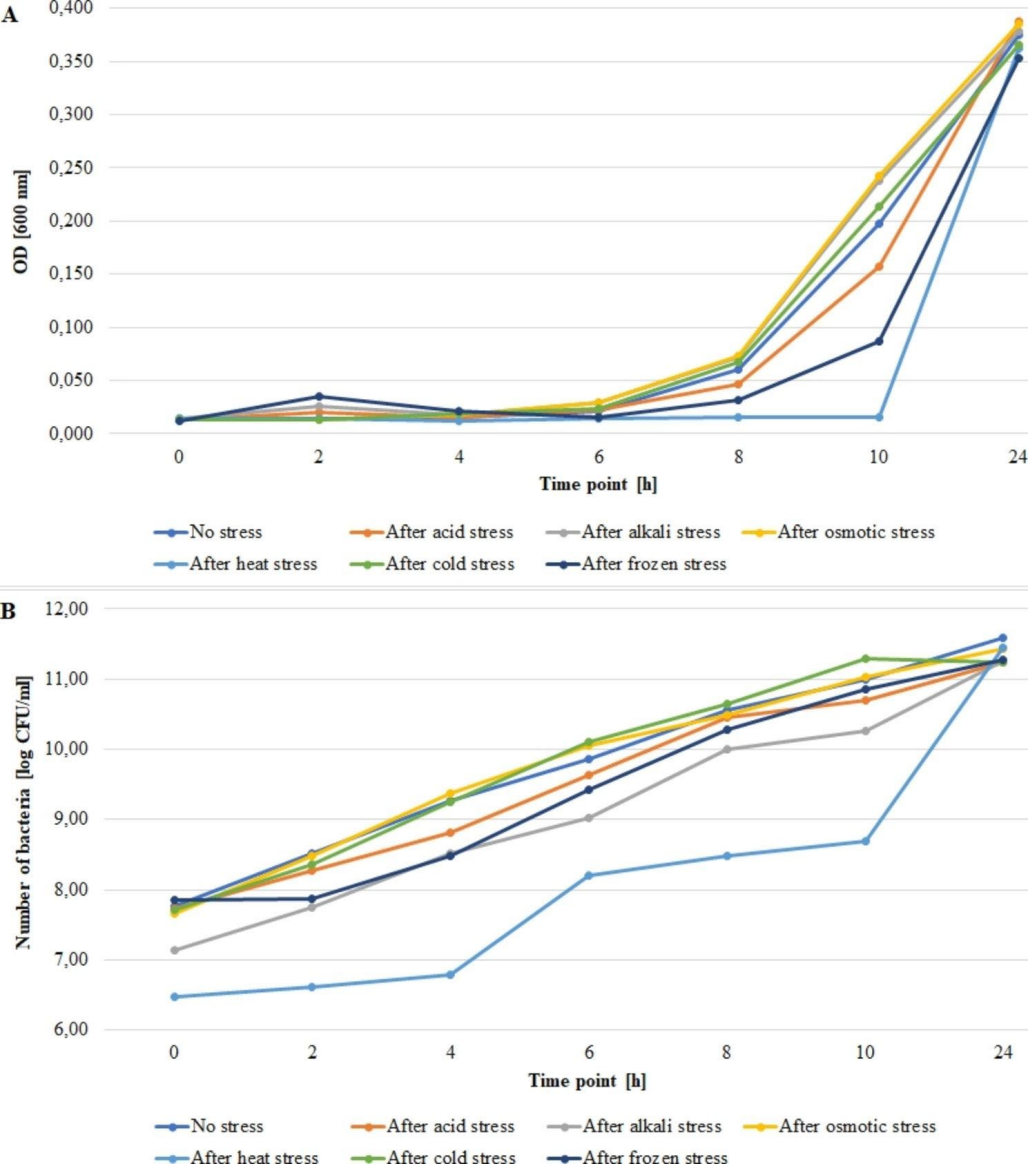
Growth rates for the no-stress and post-stress variants for strain ATCC 19111. (**A**) OD value, (**B**) number of bacteria

Figures 1, 2 and 3 present growth rates for the tested strains subjected and not subjected to stress. The average absorbance (A_600_) value at the zero point for strain 55 C ranged from 0.013 to 0.029 for frozen and acid stress, respectively (Fig. 1A). The bacteria number ranged from 6.40 to 8.26 log CFU/ml for the heat stress and no stress variants, respectively (Fig. 1B). For strain 472CC, the average value of absorbance (A_600_) at the zero point ranged from 0.011 to 0.034 for no stress variant and acid stress, respectively (Fig. 2A). The numbers of bacteria ranged from 6.90 to 7.87 log CFU/ml for heat stress and no stress variant, respectively (Fig. 2B). For strain ATCC 19111, the average absorbance value (A_600_) at the zero point ranged from 0.012 to 0.041 for frozen stress and no stress variant, respectively (Fig. 3A). The numbers of bacteria for strain ATCC 19111 at time zero ranged from 6.47 to 7.85 log CFU/ml for heat and frozen stress, respectively (Fig. 3B).

All strains exhibited the slowest growth rates after heat stress exposure. However, after 24 h of incubation, the number of bacteria after heat stress was comparable to no stress varaint. However, for both 55 C and 472CC number of bacteria after 24 h in all variants was lower than in no stress and heat stress varaints.

### Gene expression

The impact of stress exposure on *sigB*, *agrA, agrB, lmo2230* and *lmo0596* transcripts level was assessed. The expression levels of selected genes after exposure to stress were evaluated against the no stress variant.

**Fig. 4:**
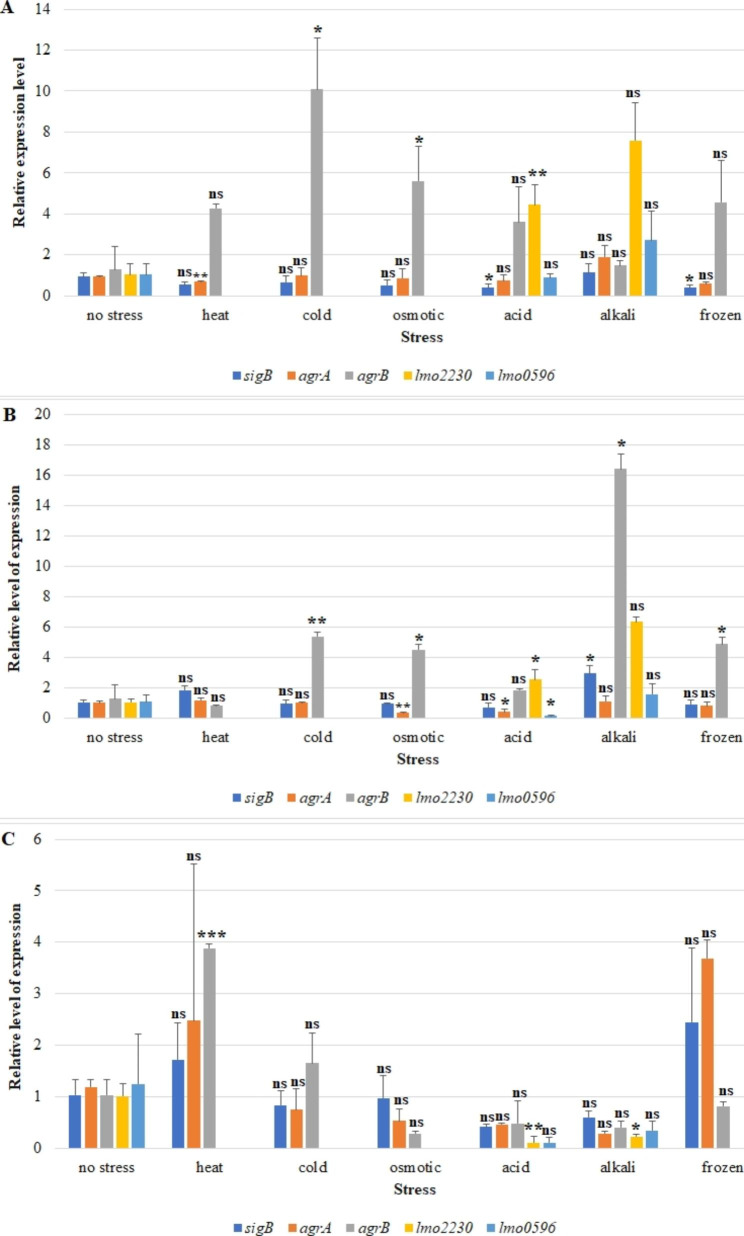
Relative level of expression of *sigB*, *agrA, agrB, lmo2230* and *lmo0596* genes for strains: (**A**) 55 C; (**B**) 472CC; (**C**) ATCC 19111. Statistical differences between experimental groups and “no stress” (reference) group were represented by appropriate symbols: *** - p < 0.001; ** - p < 0.01; * - p < 0.05; ns (no significant) - p > 0.05

The 55 C strain showed decreased *sigB* gene expression after exposure to acid (p = 0.024) and frozen (p = 0.012) stress. In contrast, higher levels of *agrB* gene expression were shown after exposure to cold (p = 0.018) and osmotic (p = 0.042) stress, and acid stress resulted in a statistically significant expression of the *lmo2230* gene (p = 0.004) (Fig. 4A). Strain 472CC displayed increased sigB gene expression after exposure to alkali stress (p = 0.028). In contrast, statistically significantly lower agrA gene expression was shown after exposure of strain 472CC to acid (p = 0.012) and osmotic (p = 0.006) stress. There was a statistically significant increase in agrB gene expression after exposure to cold (p = 0.006), osmotic (p = 0.028), alkali (p = 0.028) and frozen (p = 0.018) stress. On the other hand, acid stres significanty reduced lmo0596 gene expression (p = 0.048) (Fig. 4B).The reference strain increased agrB transcript after exposure to heat stress (p = 0.0006). There was also a decrease in lmo2230 gene expression after exposure to acid (p = 0.002) and alkali (p = 0.012) stress (Fig. 4C).

## Discussion

Survival of *L. monocytogenes* in the food production environment can lead to food contamination and epidemic outbreaks. A key factor helping bacteria survive under unpropitious environmental conditions is biofilm formation [40]. Researchers have shown that many factors, including temperature, time, type of surface, origin and availability of nutrients affect biofilm formation ability [41, 42]. Bonsaglia et al. [43] have noted that almost all strains of *L. monocytogenes* isolated from the food production environment were able to form a biofilm on stainless steel and glass. In our study, all strains produced biofilm at different levels, i.e., No. 55 C and 472CC were classified as moderate biofilm producers, and ATCC 19111 as a weak biofilm producer. Di Ciccio et al. [11] have shown that among the studied *L. monocytogenes* population, 58.0%, 38.5%, and 3.5% of the strains displayed weak, moderate or strong biofilm formation capacity, respectively. There were no differences in biofilm production depending on the isolation source [11]. In contrast, Barbosa et al. [44] have found more frequently moderate biofilm-forming ability among food than clinical strains (37 °C, 24 h conditions). In our study, strains changed a biofilm formation ability after exposure to stress factors. The strain 472CC became a strong biofilm producer (from moderate) after exposure to most stress factors except heat and acid stress. The reference strain, a weak biofilm former, produced moderate biofilm after exposure to most stressors, except for cold and frozen stress. In turn, the clinical strain, a moderate biofilm producer, formed strong and weak biofilm after cold stress and frozen stress, respectively. On the contrary, Ben Slama et al. [45] and Miladi et al. [46] have revealed that exposure to frozen stress increased ability to form biofilm of *L. monocytogenes* strains. Scientists have documented that cold shock associated with a sudden drop in temperature increased the adhesion of *L. monocytogenes* to abiotic surfaces [45, 47, 48]. Melian et al. [8] have shown that *L. monocytogenes* strain (serotype 4b) isolated from a clinical specimen formed the strongest biofilm at 10˚C. Barbosa et al. [44] have found that exposure to sublethal acid stress conditions in strain 1592/2 decreased biofilm formation ability at 37 °C. In turn, osmotic stress did not influence this ability [44]. The impact of environmental stress on biofilm formation is heterogenous in *L. monocytogenes* population and seems to depend on the particular stressor and the strain. Strong biofilm production hinders the pathogens’ eradication, increasing the risk of food contamination and an epidemic outbreak of listeriosis.

In the current study the motility of the examined *L. monocytogenes* strains after exposure to stress factors was also assessed. All bacteria were motile before and after subjecting to selected stressors after 48 h incubation. ATCC 19111 strain subjected to heat stress was nonmotile after 24 h. Lemon et al. [49] have demonstrated that flagellar-mediated motility is critical for *L. monocytogenes* adhesion and biofilm formation on abiotic surfaces. Kragh et al. [50] have shown that the survival of *L. monocytogenes* strains in a food processing environment may depend on their motility, which is necessary for biofilm formation. In turn, Cordero et al. [51] have found that *L. monocytogenes* strains with a lower degree of motility were better adapted to cold (higher growth rate) than motile strains. Also, Di Bonaventura et al. [52] have revealed no positive correlation between the motility and biofilm-forming capacity of *L. monocytogenes*. Therefore, the role of motility in survival within the food processing environment merits further investigation.

Currently, a major concern is the identification of multi-antibiotic-resistant strains of *L. monocytogenes* leading to therapeutic difficulties worldwide [53]. Resistance patterns vary across the world, which may be related to the antimicrobials use in livestock farming [54]. It seems crucial to monitor antibiotic resistance among *L. monocytogenes* isolated from different sources and different regions of the world. In our study, changes in the MIC values of selected antibiotics after exposure to stress factors were determined. In the control variant, the examined strains of *L. monocytogenes* were sensitive to all examined antibiotics. The stress exposure increased or decreased MIC values of selected antibiotics. However, these changes did not change the antibiotic susceptibility of strains. Increased MIC values were not associated with the growth rate of bacteria. Also Al-Nabulsi et al. [20] have demonstrated changes in MIC value of antibiotics after exposure to stress factors. Researchers showed an increase in the MIC of antibiotics (resistance to ampicillin, tetracycline, doxycycline and vancomycin) after exposure to 6 and 12% NaCl, pH 5 and 10˚C [20]. Alonso-Hernando et al. [55] have also shown an increase in resistance to selected antibiotics (streptomycin, cephalotin, chloramphenicol) after changing the pH of the environment with sodium hypochlorite (disk diffusion screening). Al-Nabulsi et al. [20] showed a tendency for antibiotic resistance to increase with decreasing pH. In contrast, Faezi-Ghasemi and Kazemi [56] have found that *L. monocytogenes* exposed to pH 5 or osmotic stress (7% NaCl) was more sensitive to tetracycline, rifampicin, gentamicin, penicillin, ampicillin, trimethoprim-sulfamethoxazole and chloramphenicol. In our study, the effect of osmotic and acid stress on MIC values depended on the antibiotic used and strain. There was no effect on penicillin MIC. In the case of other antibiotics, depending on the strain, a decrease, increase, or no change, were noticed. The MIC most often increased for the reference strain and 472CC. In contrast, Al-Nabulsi et al. [20] showed higher resistance for the strain isolated from meat and dairy than for the reference strain. Interestingly, heat stress decreased the MIC value of all tested antibiotics for the clinical strain and the strain isolated from cold cuts. The 55 C and 472CC strains showed a lower growth rate after exposure to heat stress. But after 24 h of incubation, the number of bacteria in the no stress variant and after heat stress exposure was comparable. The changes in the MIC values of antibiotics may result from the induction of stress shock proteins, reduction of antibiotic binding sites in the cell wall, amplification of genes responsible for synthesis and the action of the efflux pump [57]. The influence of stress factors on *L. monocytogenes* susceptibility is very variable. The demonstrated changes in the MIC values of antibiotics after exposure to the agents indicate the need to monitor the methods used to eliminate microorganisms from food processing. Inadequate procedures can increase antibiotic resistance among *L. monocytogenes* strains leading to therapeutic difficulties in patients with confirmed listeriosis.

We evaluated the growth rate of tested strains after exposure to stress factors compared to normal conditions. All tested strains showed lower growth rates after exposure to heat stress compared to the non-stress variant. Similarly Vasseur et al. [58] have observed that heat shock (55 or 63 °C, 30 min.) increased lag phase of bacteria. In our study, selected stress factors contributed to higher growth rates, i.e., for strain 55 C after exposure to osmotic, acid and alkali stress, for strain 472CC after alkali stress, for ATCC 19111 after cold stress to the selected time point. Whereas Vasseur et al. [58] have noticed that cold stress (30 min., 0 °C) had a limited effect on growth parameters. Cheroute-Vialette et al. [59] have reported that *L. monocytogenes* cells quickly overcome alkaline stress, while acid and osmotic shocks significantly changes growth parameters. In turn, Vasseur et al. [58] have revealed a decrease in growth rate with high pH values, and these changes were strain dependent.

Combining the biofilm phenotype with molecular data (gene expression levels) may provide a better understanding of the mechanism of biofilm formation by *L. monocytogenes*, especially in changing and stressful environmental conditions, such as exposure to disinfectants or nutrients deficiency [60, 61]. One mechanism helping *L. monocytogenes* to survive adverse conditions relies on alternative factor σ^B^. σ^B^ controls general stress response in *L. monocytogenes* [22, 62]. Scientists have described overexpression of *sigB* after exposure to, e.g., acid osmotic and cold stress [63–65]. Argudes-Villa et al. [66] have shown statistically significant differences in *sigB* expression between the cold-tolerant and the cold-sensitive strain. Cabrita et al. [64] have noticed higher levels of *sigB* transcript among surviving strains of *L. monocytogenes* than in sporadic strains after exposure to cold and osmotic stress. Lee et al. [23] have found that σ^B^ plays a significant role in biofilm formation under stress conditions (such as 6% NaCl, low temperature and nutrient deficiency). Researchers also showed that the wild-type strain of *L. monocytogenes* and the Δ*sigB* mutant produced very weak biofilm under stress conditions (9% NaCl, 15 °C) [23]. However, the number of viable cells for the wild type strain was significantly higher than for the Δ*sigB* mutant [23]. Essential role in the first step of biofilm formation plays the Agr system [37]. Melian et al. [8] have demonstrated overexpression of the *agrA* gene for all examined strains in the biofilm structure compared to planktonic cells. Researchers have shown an increased *agrB* gene expression in response to biofilm treatment with bacteriocin [8]. Also, Gandra et al. [67] have found an increased level of *agrA* transcripts among strains capable of biofilm formation. In turn, Rieu et al. [37] have demonstrated that the *argB* gene regulation is not dependent on the growth phase during planktonic growth. The authors have found [37] a significant decrease in *agrB* transcript levels after initial surface attachment. In contrast, Cui et al. [68] have shown lower expression levels of the genes: *agrA*, *agrC* and *agrD* (73.3%, 67.9% and 47.8%, respectively) and higher of the *agrB* (23.1%) in *L. monocytogenes* treated with cold nitrogen plasma. In our study, the *sigB* transcript level increased under alkaline (472CC) stress and the *agrB* expression increased under cold, osmotic (strains: 55 C, 472CC) alkaline and frozen stress (strain 472CC). In contrast, *sigB* transcript level decreased in response to acid and frozen stress (55 C), and *lmo2230* transcript level after exposure to acid and alkali stress (ATCC 19111). Wu et al. [69] have revealed lower transcription of the *lmo2230* gene in the Δ*sigB* strain. In addition, they [69] have shown that *lmo2230* transcription in strains from different clonal complexes differed slightly during the exponential phase, while it reached similar levels after adaptation to acid. Cortes et al. [70] have noted that the *lmo2230* gene was strongly up-regulated during lactic acid stress. In contrast, Stasiewicz et al. [71] have observed reduced or slightly elevated *lmo2230* expression after exposure of *L. monocytogenes* to organic acid salts, sodium diacetate and potassium lactate. In turn, Tessema et al. [72] have shown differential expression levels of *lmo2230* in broth adjusted to pH 5. The differences in the expression levels of the *lmo2230* gene in different studies, as in our study, can be explained by various sampling time points. In our study the *lmo0596* transcript level decreased after exposure to acid stress (ATCC 19111). In contrast, Guerreiro et al. [38] have reported that *lmo0596* transcript levels increased after exposure to acid stress in the wild-type strain, while transcript levels did not change in the Δ*sigB* strain. The gene expression may vary depending on environmental conditions, serogroup, physiological state (planktonic form vs. biofilm) and strain [73, 74]. The ability of *L. monocytogenes* to form a biofilm is a threat in the food processing environment. Therefore, it is highly relevant to explore the mechanism of stress-induced biofilm formation by *L. monocytogenes*.

Further research on a large *L. monocytogenes* population is needed to assess the molecular mechanism responsible for the correlation of antibiotic resistance, biofilm formation and resistance to stress factors (global picture of gene expression).

### Limitation of study

A limitation of this study was the small size of the study group (three strains of *L. monocytogenes*). Another limitation was reduced number of determiannts for gene expression study.

## Conclusions

We demonstrated changes in biofilm-forming capacity and MIC values of antibiotics after exposure to stress factors. These changes were strain-dependent and stressor-dependent. Changes in phenotypic characteristics after exposure to stress factors may involve increased virulence of *L. monocytogenes*, higher adaptability and survival, posing serious threat for public health. Knowledge of the changes at phenotypic level, should help planning disinfection procedures in the food industry or medical care facilities. Since changes in the antibiotic resistance profile or biofilm formation may be an individual feature of a given strain, studies on a larger population are recommended.

## Data Availability

The datasets used and/or analysed during the current study available from the corresponding author on reasonable request.

## Abbreviations

AMP: Ampicillin
ATCC: American Type Culture Coletion
BHI: Brain Heart Infusion
C: Clinical, CC:cold cuts
CAB: Columbia agar supplemented with 5.0% sheep blood
CFU: Colony forming unit
E: Erythromycin
EFSA: European Food Safety Authority
EPS: Extracellular polymeric substances
EUCAST: The European Committee on Antimicrobial Susceptibility Testing
LIPI-1: Listeria Pathogenicity Island 1
MEM: Meropenem
MHB: Mueller Hinton Broth
MHF medium: Mueller Hinton Agar with 5.0% horse blood and 20 mg/L β-NAD
MIC: Minimum inhibitory concentration
P: Penicillin
PBS: Phosphate-buffered saline
PCR: Polymerase chain reaction
R: Resistance
RPM: Revolutions per minute
RTE: Ready-to-eat
S: Sensitive
STX: Co-trimoxazole (sulfamethoxazole + trimethoprim)
TSA: Trypticase soy agar
TSB: Tryptic Soy Broth
